# A study of HBV infection and its risk factors in pregnant women in Zakho city, Iraq

**DOI:** 10.1371/journal.pone.0273362

**Published:** 2022-08-23

**Authors:** Fatima K. Khalid, Narin A. Rasheed, Nawfal R. Hussein, Ibrahim A. Naqid

**Affiliations:** 1 Department of Biomedical Sciences, College of Medicine, University of Zakho, Zakho, Kurdistan Region of Iraq, Iraq; 2 Department of Medical Microbiology, College of Medicine, University of Duhok, Duhok, Kurdistan Region of Iraq, Iraq; University of the Witwatersrand, SOUTH AFRICA

## Abstract

**Background:**

Despite vaccine and antiviral treatment availability, hepatitis B virus (HBV) continues to circulate among pregnant women in Iraq. HBV spread is due to many factors. This study evaluated HBV prevalence among pregnant women and Syrian refugees residing in Zakho city, Iraq, and identified risk factors associated with infection.

**Materials and methods:**

Demographic data were collected from 2,054 pregnant women via a questionnaire assessing risk factors associated with HBV infection. Blood samples were collected for hepatitis B surface antigen (HBsAg) and were screened using an enzyme-linked immunosorbent assay.

**Results:**

Tests revealed that 1.1% of pregnant women in Zakho and 11.3% of Syrian refugees (*p =* 0.001) were positive for HBsAg. The average age of HBsAg-positive patients was 31.4286 ± 6.6746 years (*p =* 0.002). Average rates of parity and abortion in HBV-infected subjects were 3.5 ± 2.6874 and 0.1785 ± 0.5479, respectively (*p =* 0.044 and 0.012, respectively). The following were identified as associated risk factors for HBV infection: nationality, (Zakho versus the city centre), tattoos, and polygamy (*p* = 0.001, 0.03, 0.007, and 0.001, respectively). No significant associations between HBV prevalence and blood transfusion, prior injection history, dental procedures, or surgical procedures were found.

**Conclusion:**

The prevalence of HBV infection among Syrian refugees was higher than that of indigenous participants. Several risk factors were significantly associated with HBV positivity, which may facilitate effective preventive program implementation and decrease mother-to-child transmission risk. This will likely reduce infant and childhood HBV chronicity, and mortality rates.

## 1. Introduction

Hepatitis B, caused by the hepatitis B virus (HBV), is a major global public health concern [1, 2], and leads to life threatening, chronic infections that could potentially lead to cirrhosis and hepatocellular carcinoma [1, 3]. In 2019, according to WHO, it was estimated that approximately 296 million people were living with chronic hepatitis B, and 1.5 million new infections occur annually. In the same year, approximately 820,000 deaths were recorded, mostly due to hepatitis B complications. Available and effective vaccines prevent HBV infection [1]. Hepatitis B is endemic to Iraq, with a reported prevalence ranging from approximately 1% in the northern region to 3.5% in the southern region [4–8].

Wars and human crises contribute to infectious disease spread among various regions and countries, in turn leading to changes in the global infection map [9]. Northern cities of Iraq received thousands of refugees that fled from areas experiencing conflict to neighbouring countries during the Syrian war [10, 11]. A previous study revealed that the prevalence of HBV was 3.86% among pregnant refugees, a rate higher than that of the host community of Duhok (1.09%) [9].

Hepatitis B virus can be transmitted through exposure to infected blood products and body fluids. In particular, transmission can occur via penetration such as tattooing, haemodialysis, surgery, dental procedures, and vertical transmission from mother to child [1, 4, 12]. Sexual transmission occurs most frequently among unvaccinated individuals, those with more than one partner [1], or homosexual individuals [4]. Moreover, infants delivered by mothers with hepatitis B surface antigen (HBsAg) positivity at birth or via postnatal exposure to infected blood, especially in the first 5 years of life, are at high risk of acquiring hepatitis B infection [1, 13]. Approximately 90% of these cases are at high risk of developing chronic hepatitis B in the absence of prophylactic treatment, and about 25% of chronically infected infants will die prematurely from complications of HBV including liver failure, liver cancer, and cirrhosis [13]. Moreover, most chronic infections occur in infants and in children.

Thus, in 1988, it was recommended by the Advisory Committee on Immunisation Practices (ACIP) that all pregnant women be tested for HBsAg during each gestation to ensure that babies delivered by infected mothers receive post-exposure prophylaxis, which prevents 95% of perinatal HBV infections [13, 14]. Treatment received in the third trimester of pregnancy reduces the viral load, and subsequently, reduces infection risk in the event that the infant is exposed to infected blood [15]. In addition, exposed infants should receive prenatal post-exposure prophylaxis within 12 h of birth. This critical regimen consists of hepatitis B vaccine and hepatitis B immune globulin [14]. To better prevent HBV transmission from mothers to babies, and reduce the chronicity of infection, an improved understanding of factors that increase transmission rates is needed. Hence, this study aimed to identify risk factors for HBV infection among pregnant women in the Kurdistan region of Zakho city, Iraq.

## 2. Materials and methods

### 2.1 Area of study

Zakho City is in the northern region of Iraq, on the Turkish border called Ibrahim Khalil. The distance between the border and the city centre of Zakho is 10 km. In comparison, the distance between the city centre of Zakho and the Syrian border (Peshkhabor) is 28 km. Additionally, Zakho is 58 km from the city centre of Duhok. Owing to its geographical location near the Turkish border, Zakho City acts as an intermediate station for refugees travelling to Western countries via the Turkish border; therefore, the area hosts thousands of internally displaced people and refugees.

### 2.2 Study design

This cross-sectional study was conducted between May 2019 and May 2021 in Zakho City, which is located in the Kurdistan Region of Iraq. The study aimed to recruit all the patients attending Zakho Gynecology and Obstetrics Hospital and antenatal care units in Zahko city during the study period. Thus, all pregnant women who visited the aforementioned places and agreed to participate in the study were enrolled.

### 2.3 Data collection

A questionnaire designed to collect personal data from 2,054 pregnant participants residing in the Zakho City and Syrian refugees in the Zakho City, Iraq was administered. It included questions regarding various potential risk factors of hepatitis B infection such as age, parity, nationality, Zakho Centre, history of blood/ blood product transfusion, history of surgeries, dental history, tattoos, polygamous husband, history of abortion and history of previous injections which were side-clinic based injections (Table 1).

**Table 1:** Risk factors for HBV infection.

Variables	Category	Number (n)	Percentage (%)
**Age**	14–22	556	27.069
	23–31	907	44.157
	32–40	546	26.582
	≥41	45	2.19
**Parity**	0–4	1668	81.20
	5–9	366	17.818
	10–13	20	0.973
**Abortion**	0	2002	97.468
	1	35	1.703
	2	12	0.584
	3	1	0.048
	4	4	0.194
**Nationality**	Iraqi	2001	97.41
	Syrian	53	2.58
**Living environment**	City	1292	62.9
	Rural	762	37.1
**Marriage type**	Polygamous	41	1.996
	Monogamous	2013	98
**Blood transfusion**	Yes	454	22.1
	No	1600	77.9
**Injections**	Yes	1346	65.53
	No	708	34.47
**Dental procedures**	Yes	1327	64.61
	No	727	35.39
**Surgical procedures**	Yes	170	8.276
	No	1884	91.72
**Tattoo(s)**	Yes	90	4.38
	No	1964	95.62

### 2.4 Laboratory investigations

#### 2.4.1 Sample collection

After obtaining a signed informed consent from the volunteers, they were asked to complete the study questionnaire. Blood samples (approximately 5 ml of blood) were subsequently collected from each pregnant participant. Serum samples were obtained from the blood samples via centrifugation at 1500 rpm for 3 min, and were then stored at -20°C for serological testing. Worth mention, the practical work was conducted at Zakho Gynecology and Obstetrics Hospital laboratories.

#### 2.4.2 Enzyme-linked Immunosorbent Assay (ELISA)

To detect the presence of HBsAg, serum samples were subjected to an ELISA test using a commercial HBsAg ELISA kit (ELISA 480 Test; AVONCHEM, Cheshire, UK) and ELISA 96 microwells plates. First, the anti-HBsAg antibody was applied and fixed to microwells. Subsequently, the sera of the participants was added to the fixed anti-HBsAg antibodies. After incubation, plates were washed to remove any components of the sera that were not bound to the antibodies. Secondary conjugated monoclonal antibodies bound to horseradish peroxidase were then added to the microwells of plates. After incubation, the unbound antibodies and enzymes were washed away. The stop solution and a coloured substrate were added to the wells, and the results were recorded through an ELISA reader. The concentration of antigen in a sample is calculated using the optical density (OD). Thus, as per the manufacture’s instructions, the cut off of HBsAg results are as following: a. sample (OD) / cutoff value (S/C.O) ≥1 = positive; b. sample (OD) / cutoff value (S/C.O) <1 = negative.

### 2.5 Statistics

Data analysis was performed using the Minitab 17 software, with an aim to determine whether HBV prevalence in pregnant women was associated with any of the potential risk factors. Results are presented as percentages, means ± standard deviation (SD), odds ratio (OR), and confidence intervals 95% (CI) for both study groups. The chi-square test was used to determine whether categorical data were associated with HBV prevalence, and binary logistic regression was utilised when the outcome data were continuous (age and parity). Statistical significance was set at *p* < 0.05.

### 2.6 Ethical statement

This study was approved by the Scientific and Ethics Committee of the College of Medicine, University of Zakho, Iraq (letter issued number 2019/2212). Furthermore, permission was obtained from the Zakho Gynecology and Obstetrics Hospital and Antenatal Care Unites for collecting samples and conducting practical work there. All study participants provided written consent for the use of their samples and demographic data for research purposes prior to data collection.

## 3. Results

### 3.1 Demographic data

The patient characteristics including the potential risk factors for HBV infection were assessed via a questionnaire. Findings are presented in Table 1.

### 3.2 HBV positivity

Among the 2054 participants, 28 tested positive for HBV (1.36%). The prevalence of HBV positivity among pregnant women in Zakho community was 1.1%. In comparison, among pregnant Syrian women residing in Zakho City, the prevalence of HBV was 11.3%, significantly higher than that in the Zakho community (*p =* 0.001) (Table 2).

**Table 2:** Risk factors for HBsAg positivity among pregnant women.

Factors		Infected, N (%)	Non-infected, N (%)	*p-* value	CI	OR
**Age (mean ± SD)**	----------	31.4286 ± 6.6746	27.4413 ± 6.6833	0.002	1.0313–1.1524	1.1
**Parity (mean ± SD)**	-----------	3.5 ± 2.6874	2.5657± 2.2957	0.044	1.0108–1.3322	1.16
**Abortion (mean ± SD)**	-----------	0.1785± 0.5479	0.0361± 0.2670	0.012	1.4143–16.5750	4.841
**Nationality**	Iraqi	22 (1.1)	1979 (98.9)	0.001	4.4505–29.6311	11.48
	Syrian refugee	6 (11.3)	47 (88.7)			
**Living environment**	city	12 (0.9)	1280 (99.1)	0.03	0.2057–0.9290	0.43
	rural	16 (2.1)	746 (97.9)			
**Blood transfusion**	yes	10 (2.2)	444 (97.8)	0.09	0.9073–4.3188	1.98
	no	18 (1.1)	1582 (98.9)			
**Marriage type**	polygamy	5 (12.2)	36 (87.8)	0.001	4.3257–33.3832	12.02
	monogamy	23 (1.1)	1990 (98.9)			
**Injection**	yes	18 (1.3)	1328 (98.7)	0.9	0.4344–2.0606	0.95
	no	10 (1.4)	698 (98.6)			
**Dental procedures**	yes	19 (1.4)	1308 (98.6)	0.7	0.5216–2.5747	1.16
	no	9 (1.2)	718 (98.8)			
**Surgical procedures**	yes	1 (0.6)	169 (99.4)	0.3	0.0550–3.0135	0.41
	no	27 (1.4)	1857 (98.6)			
**Tattoo(s)**	yes	5 (5.6)	85 (94.4)	0.007	1.8424–13.3759	4.96
	no	23 (1.2)	1941 (98.8)			

HBsAg, hepatitis B surface antigen

### 3.3 Risk factors for HBV infection in pregnant women

Various risk factors for HBV infection were considered and the following factors were found to be significantly associated with the HBV infection prevalence: age, parity, abortion, nationality, Zakho centre, tattoo, and marriage type (*p* = 0.002, 0.044, 0.012, 0.001, 0.03, 0.007, and 0.001, respectively) (Table 2).

## 4. Discussion

HBV infection is a global health concern and it is an endemic infection in Iraq, with its prevalence been reported in previous studies. The prevalence rate of HBV among participants from the host community of Zakho City was 1.1%, a rate higher than the previously reported rates (0.54%) [16]. Moreover, this rate was similar to a previous report that assessed the HBV infection rate in the northern region of Iraq, but lower than values reported for the southern region of Iraq [2, 8] and Turkey (4.57%) [17]. The discrepancy among reported values may be due to differences in study sample sizes and sampling methods. To the best of our knowledge, this is the first study performed in Iraq to recruit a large number of pregnant women exclusively.

The rate of HBsAg positivity in refugee subjects was 11.3%, a rate higher than the previously reported HBsAg positivity rates among Syrian refugees in Duhok city, Iraq (3.86%) [9], Ethiopia (6.8%) [18], and refugees in Mahama, Rwanda (3.8%) [19]. Notably, these studies addressed the general population of refugees, and not exclusively pregnant women. This finding may indicate that rates of HBsAg positivity are elevated among pregnant women, a finding of great significance because high HBsAg positivity in pregnancy may lead to an increase in the prevalence of HBV in the community via the delivery of infected infants. Without closely monitoring the health of the infected infants, they are at a high risk of chronic infection, and will likely pass HBV to the next generation. Urgent plans are needed to encourage HBV screening of pregnant women in refugee camps. In addition, our results revealed that the prevalence of HBV infection among refugees was significantly higher than that reported among indigenous women. The high HBsAg positivity rates observed among refugees may be due to their limited access to the health care system, and the lack of affordable preventive programs and treatment options. Further, difficult living circumstances, social and cultural discrimination, and sexual violence may promote adverse behaviours, and consequently, enhance the HBV infection risk [18]. Increased rates of HBV infection threaten international communities, since Zakho City serves as a stopping point for refugees that later travel to a variety of Western countries via the Iraqi-Turkish border. It is worth mentioning that all the positive HBsAg cases in the study were referred to the infectious diseases and gastroenterology units for further evaluation.

Age and parity were also identified as risk factors for HBV infection. Moreover, a high degree of parity is associated with an increased risk of HBV infection. This may be due to a lack of continuous screening, lack of personal hygiene, and limited access to health services in rural areas. In addition, age was associated with an increased risk of infection. Our results are similar to previous study findings that revealed age to be a risk factor for HBV infection in Iran and China [20, 21]. This finding is likely due to the cohort effect and continual exposure to risk factors throughout life.

A history of abortion was reported to be significantly associated with HBsAg positivity. This may be due to blood or hospital-associated contamination of surgical instruments used in abortion procedures, therefor, additional studies will be needed to further investigate this aspect. Further, improved protocols that aim to prevent HBV infection in hospitals are needed.

No significant association between HBV infection and the occurrence of dental procedures was identified. Our results disagree with those of a previous study conducted in Iraq [2], but are similar to those of a study conducted among refugees in Ethiopia [18]. The discrepancy in results may be due to the recently adopted infection control measures by dentists prior to and during dental work.

Notably 2.2% and 1.1% of participants with and without a blood transfusion history were determined to be infected with HBV. However, no significant association between blood transfusion history and HBsAg positivity was observed. Our results are similar to those of previous studies conducted in the region [2], and could be attributed to the viral screening practices that are required before donating or receiving blood.

In our society, intramuscular and intravenous injections are administered outside hospitals by untrained personnel. Hence, they are considered risk factors for blood-borne viral infections. In agreement with a previous study conducted among blood donors in Iraq, [2] a history of previous injections and history of surgical procedures did not correlate with HBsAg positivity. Our findings are in contrast to previous study findings that identified a significant association between HBsAg positivity and surgical history in pregnant women [4, 21]. In contrast to previous studies [2, 4, 18], this study identified tattoo presence as a risk factor for HBV positivity. Tattooing is often performed by untrained individuals about infection control, and increased infection rates reported here may be due to the use of contaminated instruments.

In Iraq, polygamy is legal. We found that HBV infection rates were higher in women with polygamous than monogamous husbands. Our results were similar to those of a study conducted in Iran, which showed that marriage is a risk factor for HBV infection [20]. In general, having more than one partner increases infection risk, especially when participating in sexual illegitimate practices [2]. Despite the presence of a screening policy in the civil marriage process in Iraq, which states that all new couples are required to undergo viral infection screening before marriage, results of the study showed that high HBV infection rates remain a problem. Few families prefer religious marriages in mosques or churches to civilian marriages. In these cases, viral screening procedures are not required prior to marriage, thus increasing HBV infection risk and impeding HBV infection control.

The residing location of the participants was significantly associated with HBV positivity. HBV positivity rates for participants residing in the city centre of Zhakho (0.9%) was lower than that of those living in rural areas (2.1%). This may be due to the lack of knowledge regarding HBV in the rural population, and the distance between the participants’ residence and health care centres.

This is the first study to assess potential risk factors of HBV infection among a large number of pregnant women. The prevalence of HBV among Syrian refugees was significantly higher than that of participants indigenous to Zakho city, Iraq. The following risk factors are associated with HBsAg positivity: age, parity, nationality, area of residence, tattoo, marriage type, and whether the women previously had an abortion. No significant associations between HBV prevalence and blood transfusion, prior injection, dental treatment, and surgical history were identified. These findings have the potential to improve public health measures that aim to prevent mother-to-child HBV transmission. Based on our findings, we recommend the following measures:

1. All pregnant women to undergo screening pre/during pregnancy to detect HBV at an early stage and reduce perinatal transmission
2. Report all HBsAg-positive tests to the health authorities to facilitate case follow-up and infant monitoring
3. Administer antivirals to pregnant women in their third trimester to reduce the viral load, which may in turn reduce the risk of infection transmission to infants.
4. Mandatory vaccination and prenatal exposure prophylaxis, especially after birth or in the early hours after birth
5. Public education and awareness are emphasized.

## Abbreviations

ACIP: Advisory Committee on Immunisation Practices
ELISA: Enzyme-linked Immunosorbent Assay

## References

[1] World Health Organization. Hepatitis B, https://www.who.int/news-room/fact-sheets/detail/hepatitis-b

[2] R Hussein NR. Risk factors of hepatitis B virus infection among blood donors in Duhok city, Kurdistan Region, Iraq. Caspian J Intern Med 2018;9:22–6. doi: 10.22088/cjim.9.1.22 29387315 PMC5771356

[3] Abdullah RA, Badal R, Hussein NR, Jaafar S, Yaqoob ES, Salih RS, et al. Hepatitis B knowledge among healthy volunteers in Duhok City, Kurdistan region, Iraq. Int J Infect 2017;4. 10.5812/iji.14976.

[4] Khalil AS, Hussein NR, Shamdeen MY. Impact of maternal HBsAg carrier status on pregnancy outcomes in Duhok city, Iraq. Asian Pac J Trop Biomed 2017;7:1010–3. 10.1016/j.apjtb.2017.09.023.

[5] Al-Juboury AW, Salih HA, Al- Assadi MK, Ali AM. Seroprevalence of hepatitis B and C among blood donors in Babylon governorate-Iraq. Med J Babylon 2010;7:121–9.

[6] Mahmood AK, Addose SA, Salih HA, Khadi AA. Seroprevalence of HBsAg and Anti HCV positive blood donors in Najaf governorate. Iraqi Jcomm Med 2001;14:29–33.

[7] Saleem M, Sidiq Z, Naqid IA, Hussein NR, Mohammad SJ, Noaman JS, et al. The prevalence of hepatitis B and C Virus in patients with end-stage kidney disease on regular hemodialysis in Duhok, Iraq: a brief report. Avicenna J Clin Microbiol Infect 2020;7:31–3.

[8] Hussein NR, Zana ZS, Ibrahim NM, Assafi MS, Daniel S. The prevalence of HBV infection in renal transplant recipients and the impact of infection on graft survival. Acta Med Iran 2019:381–4.

[9] Hussein NR, Abdullah IM, Younus OM, Taher AM, Salim AA, Shahab FI. Prevalence of HBV, HCV and HIV infections among Syrian refugees in Kurdistan region, Iraq. Int J Infect 2017;4:e39420.

[10] Rasheed NA, Hussein NR. Methicillin-resistant Staphylococcus aureus carriage rate and molecular characterization of the staphylococcal cassette chromosome mec among Syrian refugees in Iraq. Int J Infect Dis 2020;91:218–22. doi: 10.1016/j.ijid.2019.12.006 31843670

[11] Rasheed NA, Hussein NR. Characterization of different virulent factors in methicillin-resistant Staphylococcus aureus isolates recovered from Iraqis and Syrian refugees in Duhok city, Iraq. PloS one 2020;15:e0237714. doi: 10.1371/journal.pone.0237714 32804961 PMC7430753

[12] MacLachlan JH, Locarnini S, Cowie BC. Estimating the global prevalence of hepatitis B. Lancet 2015;386:1515–7. doi: 10.1016/S0140-6736(15)61116-3 26231458

[13] Centers for Disease Control and Prevention. Laboratory reporting of pregnancy status for hepatitis B-positive women, https://www.cdc.gov/hepatitis/hbv/pregstatuslabreporting.htm; 2021.

[14] Shepard CW, Simard EP, Finelli L, Fiore AE, Bell BP. Hepatitis B virus infection: epidemiology and vaccination. Epidemiol Rev 2006;28:112–25. doi: 10.1093/epirev/mxj009 16754644

[15] Navabakhsh B, Mehrabi N, Estakhri A, Mohamadnejad M, Poustchi H. Hepatitis B virus infection during pregnancy: transmission and prevention. Middle East J Dig Dis 2011;3:92. 25197539 PMC4154922

[16] Jamal SA, Naqid IA, Hussein NR, Yousif SH, Yousif SA, Hasso SS, et al. The prevalence of hepatitis B and C Virus in healthy women in Zakho City, Kurdistan region of Iraq: a brief report. J Kermanshah Univ Med Sci 2019;23. 10.5812/jkums.99337.

[17] Özkan H. Epidemiology of chronic hepatitis B in Turkey. Euroasian j hepatogastroenterol 2018;8:73. doi: 10.5005/jp-journals-10018-1264 29963468 PMC6024053

[18] Ayele A, Abera D, Hailu M, Birhanu M, Desta K. Prevalence and associated risk factors for hepatitis B and C viruses among refugees in Gambella, Ethiopia. BMC Public Health 2020;20:1–10 doi: 10.1186/s12889-020-08893-1 32429964 PMC7236441

[19] Kamali I, Barnhart DA, Ndahimana JD, Noor K, Mumporeze J, Nyirahabihirwe F, et al. Prevalence and associated risk factors for hepatitis B and C viruses among refugee populations living in Mahama, Rwanda: A cross-sectional study. PloS one 2021;16:e0257917. doi: 10.1371/journal.pone.0257917 34634039 PMC8504757

[20] Alavian SM, Tabatabaei SV, Ghadimi T, Beedrapour F, Kafi-Abad SA, Gharehbaghian A, et al. Seroprevalence of hepatitis B virus infection and its risk factors in the west of Iran: a population-based study. Int J Prev Med 2012;3:770. 23189228 PMC3506088

[21] Li X, Zheng Y, Liau A, Cai B, Ye D, Huang F, et al. Hepatitis B virus infections and risk factors among the general population in Anhui Province, China: an epidemiological study. BMC Public Health 2012;12:1–7. doi: 10.1186/1471-2458-12-272 22475135 PMC3355038

